# P-108. Clinical Evaluation of a Sequencing-based Diagnostic for Bacterial and Fungal ID & AST Directly from Patient Blood Samples

**DOI:** 10.1093/ofid/ofae631.315

**Published:** 2025-01-29

**Authors:** Michael R Filbin, Peter Hou, Michael Donnino, Archana Asundi, Zoe H Rogers, Emma Briars, Alison Gassett, Alexander Reidel, Alexis Campbell, Jason Wittenbach, Nicole Billings

**Affiliations:** Massachusetts General Hospital, Boston, Massachusetts; Division of Emergency Critical Care Medicine, Department of Emergency Medicine, Brigham and Women’s Hospital; Harvard Medical School, Boston, MA, Boston, Massachusetts; Beth Israel Deaconess Medical Center, Boston, Massachusetts, Boston, Massachusetts; Boston Medical Center, Boston, Massachusetts; Day Zero Diagnostics, Allston, Massachusetts; Day Zero Diagnostics, Allston, Massachusetts; Day Zero Diagnostics, Allston, Massachusetts; Day Zero Diagnostics, Allston, Massachusetts; Massachusetts General Hospital, Boston, Massachusetts; Day Zero Diagnostics, Allston, Massachusetts; Day Zero Diagnostics, Allston, Massachusetts

## Abstract

**Background:**

Early pathogen ID and targeted treatment are key to reducing bloodstream infection (BSI) morbidity and mortality. Current diagnostics rely on culture which takes 1-2 days for ID and longer for antimicrobial susceptibility testing (AST) results, or molecular assays with limited panels, few resistance markers, and high false positive rates. We report interim results of a first-in-kind comprehensive ID and predictive AST assay directly from patient blood samples. The system can deliver results in ∼8 hrs, uses ultra-high enrichment of microbial DNA directly from blood, whole-genome sequencing, and a predictive AST machine-learning algorithm to identify a broad range of species and pathogen/drug combinations.

**Figure:**
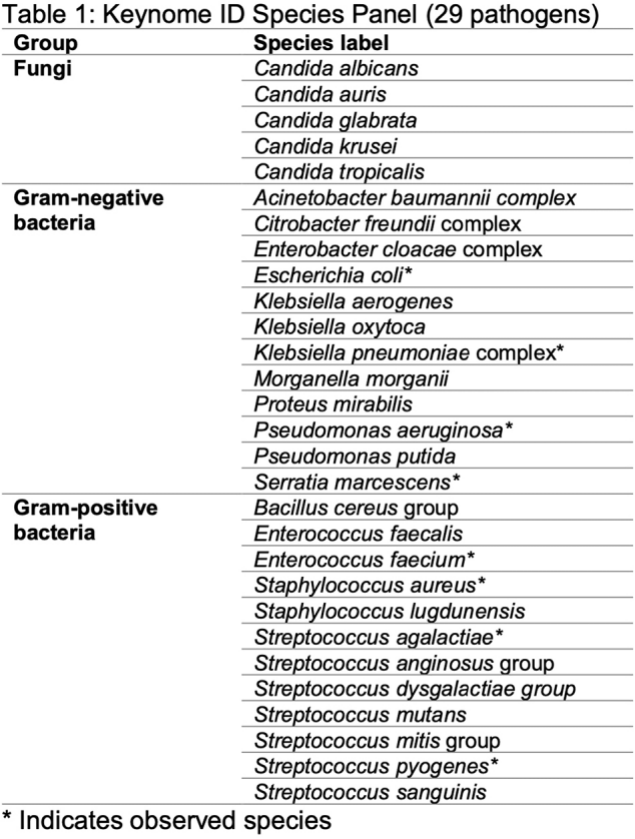

**Methods:**

We enrolled subjects with suspicion of BSI from 3 EDs and 1 ICU/inpatient in 4 Boston area hospitals in 2 IRB approved observational studies. We collected whole blood in SPS vacutainers and 10mL were processed at Day Zero Diagnostics (DZD) and sequenced on an Oxford Nanopore platform. Sequencing data were analyzed by Keynome® algorithms to determine pathogen ID and predict AST profiles. Tables 1 & 2 list current on-panel pathogens and drug models tested. Performance was compared to hospital microbiology lab phenotypic ID/AST results from blood cultures collected within 0-24 hours of the research draw.

**Figure:**
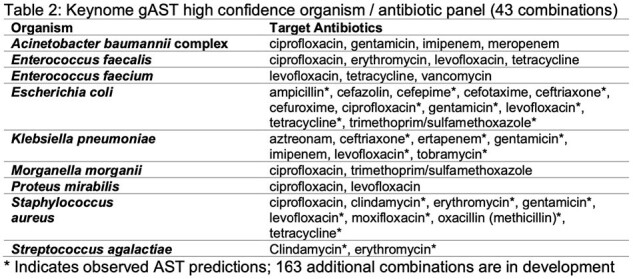

**Results:**

Species-level ID results in 225 subjects (6525 calls) demonstrated 80.0% sensitivity, 99.9% specificity, 64% PPV, and 99.9% agreement with clinical culture. Eight distinct species were identified among the 20 (8.9%) clinical culture positive samples with on-panel organisms. Thirteen samples had sufficient genome coverage for on-panel AST predictions demonstrating 92.3% categoric agreement with phenotypic AST.

**Conclusion:**

Our data suggests the DZD diagnostic system can provide accurate ID and AST results directly from blood in patients with suspected BSI. To our knowledge these results are the first demonstration of whole genome recovery and comprehensive ID & AST directly from patient blood samples. This assay has the potential to revolutionize speed to diagnosis of BSI, thus facilitating targeted therapy, improved outcomes, and reduced development of antimicrobial resistance.

**Disclosures:**

**Michael R. Filbin, MD**, Day Zero Diagnostics: Grant/Research Support|Quidel: Grant/Research Support **Peter Hou, MD**, Center for Disease Control: Grant/Research Support|Day Zero Diagnostics: Grant/Research Support|iDoc Telehealth Solutions: Ownership Interest **Michael Donnino, MD**, Day Zero Diagnostics: Grant/Research Support **Archana Asundi, MD**, Day Zero Diagnostics: Grant/Research Support|Gilead Sciences: Grant/Research Support|GSK/ViiV Healthcare: Grant/Research Support|Theratechnologies: Grant/Research Support **Zoe H. Rogers, MPH**, Day Zero Diagnostics: employment **Emma Briars, PhD**, Day Zero Diagnostics: employment **Alison Gassett, MPH**, Day Zero Diagnostics: employment **Alexander Reidel, BS**, Day Zero Diagnostics: employment **Alexis Campbell, MA**, Day Zero Diagnostics: Grant/Research Support **Jason Wittenbach, PhD**, Day Zero Diagnostics: employment **Nicole Billings, PhD**, Day Zero Diagnostics: employment

